# Internet-based cognitive behavior therapy for obsessive compulsive disorder: A pilot study

**DOI:** 10.1186/1471-244X-11-125

**Published:** 2011-08-03

**Authors:** Erik Andersson, Brjánn Ljótsson, Erik Hedman, Viktor Kaldo, Björn Paxling, Gerhard Andersson, Nils Lindefors, Christian Rück

**Affiliations:** 1 Department of Clinical Neuroscience, Division of psychiatry, Karolinska Institutet, Stockholm, Sweden; 2 Department of Epidemiology and Biostatistics, EMGO Institute for Health and Health Care Research, VU University Medical Centre, Amsterdam, Netherlands; 3 Department of Behavioural Sciences and Learning, Swedish Institute for Disability Research, Linköping University, Linköping, Sweden

**Keywords:** Cognitive behavior therapy, Internet, obsessive compulsive disorder

## Abstract

**Background:**

Cognitive behavior therapy (CBT) is widely regarded as an effective treatment for obsessive compulsive disorder (OCD), but access to CBT therapists is limited. Internet-based CBT (ICBT) with therapist support is a way to increase access to CBT but has not been developed or tested for OCD. The aim of this study was to evaluate ICBT for OCD.

**Method:**

An open trial where patients (N = 23) received a 15-week ICBT program with therapist support consisting of psychoeducation, cognitive restructuring and exposure with response prevention. The primary outcome was the Yale-Brown Obsessive Compulsive Scale (Y-BOCS), which was assessed by a psychiatrist before and immediately after treatment. Secondary outcomes were self-rated measures of OCD symptoms, depressive symptoms, general functioning, anxiety and quality of life. All assessments were made at baseline and post-treatment.

**Results:**

All participants completed the primary outcome measure at all assessment points. There were reductions in OCD symptoms with a large within-group effect size (Cohen's *d *= 1.56). At post-treatment, 61% of participants had a clinically significant improvement and 43% no longer fulfilled the diagnostic criteria of OCD. The treatment also resulted in statistically significant improvements in self-rated OCD symptoms, general functioning and depression.

**Conclusions:**

ICBT with therapist support reduces OCD symptoms, depressive symptoms and improves general functioning. Randomized trials are needed to confirm the effectiveness of this new treatment format.

**Trial Registration:**

ClinicalTrials.gov: NCT01348529

## Background

Obsessive compulsive disorder (OCD) is a chronic condition characterized by obsessions and/or compulsions (e.g. fear of dirt, need for symmetry, compulsory checking) [1]. OCD is associated with lowered quality of life, social isolation [1] and a large economic burden on society [2]. The lifetime prevalence is estimated to be 2-3% [3]. First line treatments are serotonin reuptake inhibitors and cognitive behavior therapy (CBT) [4]. Although there is substantial evidence for the effectiveness of CBT [5], there is generally limited access to CBT therapists [6,7]. Therefore, as evidence-based psychological treatment is scarce, it is essential to develop more time- and cost-efficient treatments for OCD patients who do not receive CBT. Self-help treatments have the potential to reduce therapist time and make the treatment more accessible to patients [8]. Self-help treatments with therapist support has been found to be equally effective as face-to-face treatments [9] whereas studies without the therapist have lower effects [10].

Self-help treatments for OCD have been developed and evaluated previously [11-14] with medium to large effect sizes. One specific type of self-help treatment is computer-based CBT. In a small study by Clark et al. [15], a computer program was tested for OCD patients with checking and washing rituals. The program included interactive animations to model exposure with ritual prevention (ERP) and resulted in reduced washing and checking rituals [15]. Another example of a computer-based treatment for OCD is "BT-steps" [16], which includes education, assessment, and self-exposure techniques. In BT-steps, the patient works with a self-help book and reports progress with the treatment using an automated phone interactive-voice-response. BT-steps has been evaluated in four different trials [17-20]. The results from these trials show that computer-based CBT is effective for reducing OCD symptoms but that the treatment format may be inferior to traditional CBT, mainly due to higher dropout rates. One of the studies [20] also shows that dropout rates decreases if telephone support is added. Hence, therapist support appears beneficial in this treatment format which is in line with what has been observed for several other conditions [21].

CBT can be delivered via the Internet. Internet-based CBT (ICBT) with therapist support is a type of computerized treatment in which the patient logs onto a website and works with written self-help material and homework assignments [21,22]; the patient's work with the treatment is supported by regular contact with an online therapist. One advantage of ICBT is that the text material, work sheets, self-rating questionnaires and therapist contact are integrated into a single system. The main function of the therapist is to provide support through clarifying information, monitoring progress, giving feedback on homework assignments and allowing the patients access to the sequential treatment steps. ICBT has been effective in randomized controlled trials on a wide range of psychiatric and medical problems [23-25], and has the advantage of saving therapist time compared to traditional treatment. Therefore, ICBT has the potential to be a cost-effective alternative for OCD and increases treatment accessibility [26].

Previous self-help trials on OCD have been strictly computer-based and/or have featured very limited therapist support. This is important as evidence suggest that self-help treatments are more effective when guided by a therapist [21]. There are, to our knowledge, no published data on ICBT with therapist support in the treatment of OCD. Therefore, an open pilot study was conducted to evaluate the value of ICBT with therapist support for OCD before moving on to a controlled trial. A treatment program for OCD was developed in which patients receiving treatment were expected to improve on measures of OCD, global functioning, quality of life and secondary psychiatric symptoms.

## Method

### Participants

The study was approved by the regional ethics committee in Stockholm. Informed written consent was obtained from all study participants. Participants were recruited in Stockholm by referral from primary care physicians, mental health professionals and self-referral. Information about the study was published on the official web page of the clinic http://www.internetpsykiatri.se.

To be eligible for inclusion, the following criteria had to be fulfilled by the participants: (a) to agree not to undergo any other psychological treatment for the duration of the study; (b) to have no history of CBT for OCD during the last two years; (c) to fulfill the Diagnostic and Statistical Manual of Mental Disorders 4th ed. (DSM-IV) [27] criteria of OCD according to the structured clinical interview for mental disorders (SCID-I) [28]; (d) to have no serious physical illness; (e) to have constant dosage two months prior to treatment if on prescribed psychotropic medication, and agree to keep dosage constant throughout the study; (f) other comorbid disorders according to the Mini International Neuropsychiatric Interview (MINI) [29] were acceptable but the OCD diagnosis had to be primary; (g) no alcohol or drug abuse dependency; (h) to have no history of psychosis or bipolar disorder, (i) Yale-Brown Obsessive Compulsive Scale (Y-BOCS) < 31 which is the established cut-off for extreme OCD [30] and (j) OCD symptoms not primarily associated with hoarding. The reason for the hoarding exclusion criterion was that the treatment of hoarding differs substantially from other OCD-related symptoms [31] and in the forthcoming DSM-V [32], hoarding is proposed as a separate disorder. Therefore, the inclusion of hoarders would potentially decrease future ecological validity and clinical usefulness of this study.

**Figure 1:**
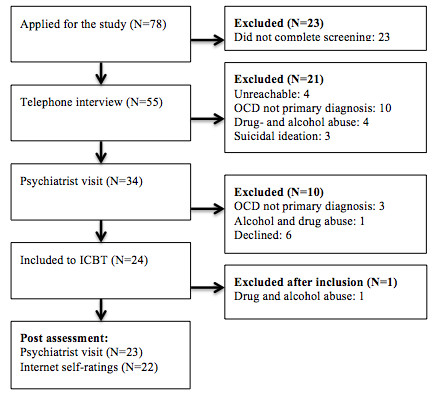
**Flowchart**.

In the first stage of the recruitment process, participants conducted an Internet screening consisting of the Y-BOCS (self-rating version) [33], Obsessive Compulsive Inventory - Revised (OCI-R) [34], Montgomery Åsberg Depression Rating Scale - Self report (MADRS-S) [35], Alcohol Use Disorders Identification Test (AUDIT) [36], and the Drug User Disorders Identification Test (DUDIT) [37]. In the next step, participants were interviewed over the telephone to determine whether they met inclusion criteria a and b; obvious cases of non-OCD were excluded in this step (e.g. patients with a primary diagnosis of trichotillomania). Of the 55 individuals screened, 34 individuals continued to a face-to-face diagnostic interview with a psychiatrist (independent assessor) who had extensive training in OCD diagnostics. Through the interview, it was determined whether criteria c-j were fulfilled and OCD severity was assessed with a clinician administered Y-BOCS [30]. Twenty-four of the interviewed participants fulfilled all criteria and were included in the study. Two weeks after inclusion, one participant reported serious problems with drug and alcohol abuse which had not been reported at the psychiatrist visit. This participant was retrospectively excluded from the study and all data analyses. All 23 participants completed primary outcome assessment with a psychiatrist at baseline and post-treatment. All participants except one also completed the self-rated outcome measures. The participant flow throughout the trial is displayed in Figure 1 and the demographic description of the participants is presented in Table 1. Therapist time (i.e. time to read the reports and write feedback) was logged automatically in the treatment platform. The therapist was not allowed to do any patient-related work (writing feedback etc.) outside the treatment platform log system.

**Table 1:** Demographic description of the participants

Variable		Treatment cohort	
		N	%

**Gender**	Women	15	65%
	
	Men	8	35%

**Age**	Mean age (SD)	39	
	
	Min-max	21-67	

**Occupational status**	Working full time	17	74%
	
	Student	2	9%
	
	Full-time sick-leave	1	4%
	
	Unemployed	2	9%
	
	Pensioner	1	4%

**Earlier psychological treatment**	Psychodynamic Therapy	5	22%
	
	Cognitive therapy	1	4%
	
	Cognitive Behavior therapy	5	22%
	
	Non-specific counseling	3	13%
	
	None	9	39%

**Education**	High school	8	35%
	
	University < 3 years	2	9%
	
	University ≥ 3 years	12	52%
	
	PhD	1	4%

**Psychotropic medication**	SSRI	4	17%
	
	Benzodiazepines	4	17%

**Psychiatric contact**	Mean length in years	1.93	
	
	Min-max	0-14	

**OCD duration**	Mean length in years	13	
	
	Min-max	4-56	

**Referral**	Self referral	13	57%
	
	GP referral	4	17%
	
	Mental health professional referral	6	26%

### Outcome measures

#### Diagnosis and global functioning

The participants underwent a structured diagnostic interview, in which SCID [28] was used for OCD and MINI [38] for other diagnoses. The SCID has acceptable reliability with Cohen's Kappa coefficients ranging .70-1.00 [28] and the MINI has high diagnostic concordance with other diagnostic tools [38]. The reason for conducting the SCID on OCD criteria was that the SCID provides more detailed questions than the MINI. The Clinical Global Impression Scale (CGI) [39] and Global Assessment of Functioning (GAF) [27] were used to measure global improvement. Although not previously validated for an OCD population, both CGI [40] as well as the GAF [41] have shown satisfactory reliability in other psychiatric populations. Diagnostic and global functioning was assessed during the psychiatric interview at both baseline and post-treatment.

#### Yale Brown Obsessive Compulsive Scale (Y-BOCS)

The primary outcome measure was the clinician-administered Y-BOCS [30], which is regarded as the gold standard for assessing the severity of OCD symptoms [42]. Y-BOCS comprises 10 items, rated on a 5-point Likert scale ranging from 0 (no symptoms) to 4 (severe symptoms). The total score ranges from 0 to 40 and consists of two sub scores for compulsions (range 0 to 20) and obsessions (range 0 to 20). Y-BOCS has excellent inter-rater reliability and moderate to good internal consistency [30]. There are moderate correlations between the clinician and the self-rating version of the Y-BOCS, with the obsession subscale having lower convergence between the two versions [43]. However, another study has shown a correlation between the computer and clinician administered Y-BOCS of .88 [33]. The clinician version of Y-BOCS was administered at baseline and post-treatment by a psychiatrist and administered as a self-rating questionnaire at screening, baseline and post-treatment [33].

#### Obsessive Compulsive Inventory - Revised (OCI-R)

The secondary outcome measure of OCD symptoms was the OCI-R which included 18 items measuring six different symptom dimensions [34]. The OCI-R total score has high test-retest reliability (alpha values from .81 to .89) and high sensitivity to change, in relation to the Y-BOCS [44]. The OCI-R was administered at screening, baseline, post-treatment, and weekly during treatment to monitor OCD symptoms.

#### Montgomery Åsberg Depression Rating Scale - Self report (MADRS-S)

The MADRS-S is a 9-item questionnaire assessing depressive symptoms [35]. The MADRS-S has good test-retest reliability (.80 - .94) and correlates (*r *= .87) with the Beck Depression Inventory, indicating acceptable convergent validity [45]. Previous research has shown that the psychometric properties of the MADRS-S remains unchanged after transformation to online use [46]. The MADRS-S was used at screening, baseline, post-treatment and weekly during treatment to assess and monitor depressive symptoms.

#### Penn State Worry Questionnaire (PSWQ)

The PSWQ is a 16-item inventory designed to assess pathological worry. The PSWQ has high internal consistency and good test-retest reliability [47] and the computerised version has shown high convergence compared to the paper and stencil version [48]. The PSWQ was administered at baseline and post-treatment.

#### Euroqol (EQ-5D)

The euroqol (EQ-5D) is used as a generic measurement of global functioning and quality of life [49]. The EQ-5D is non-disease specific and measures five health domains of importance to quality of life: mobility, self-care, usual activities, pain/discomfort, and anxiety/depression [50]. The measure has good psychometric properties including acceptable test-retest reliability over 7 to 10 days (intraclass coefficient = .82 - .83) and acceptable convergent validity [51]. The computer administered EQ-5D correlates highly (.85) with the paper and pencil version [52]. The EQ-5D was administered at baseline and post-treatment.

#### Quality of life Inventory (QOLI)

QOLI measures quality of life [53] and consists of 16 items covering different life domains, such as work and health. QOLI has good test-retest reliability with alpha coefficients of .80-.91 and good internal consistency [53]. The online version of the QOLI has been compared to the paper and pencil version with mixed results [54,55]. The QOLI was administered at baseline and post-treatment.

### Treatment

The self-help manual consisted of 15 modules, comprising 100 pages. The manual was written by the first author (EA) and was inspired by a self-help book [56]. Six experienced clinical psychologists who worked at an OCD clinic read the manual, gave written feedback, and met for a three-hour focus group to discuss possible improvements of the manual. The manual was further developed after this feedback. The contents of the modules are presented in Table 2.

**Table 2:** Summary of the content of the OCD treatment manual

Module 1 *CBT and OCD explained*	Treatment rationale is presented, including a description of OCD symptoms (obsessions and compulsions), prevalence, and main principles of conducting an online CBT treatment. Different fictional patient characters are introduced (each example represents a specific OCD symptom dimension). The participant has the opportunity to follow one or all four characters (washing, checking, symmetry, or violent thoughts). *Homework: Register OCD symptoms in the Internet platform diary.*
Module 2 *Assessing OCD symptoms with* *the CBT model*	The autonomic nervous system and its interaction with OCD symptoms is explained. Participants begin to link obsessions and compulsions to the OCD circle and learn how to conduct a functional analysis of their OCD problems. Each OCD circle is presented visually for each example character. *Homework: Continue OCD diary registrations and apply these to the OCD circle.*
Module 3 *Cognitive restructuring*	Common OCD metacognitions are explained, such as inflated responsibility, absolute need for certainty, thought-action fusion and exaggerated need to control. The focus is to register and discuss meta cognitions with the psychologist from a functional perspective. *Homework: Continue OCD diary registrations and use these registrations to analyze meta cognitions associated with obsessions.*
Module 4 *Establish treatment goals and exposure hierarchy*	Introduction to Exposure with response prevention (ERP). Different strategies for conducting ERP are explained and examples given of treatment goals and different ways of constructing exposure hierarchies for each example character. *Homework: register treatment goals and then construct an exposure hierarchy with the information from these goals.*
Module 5 *Exposure with response prevention (ERP)*	Different aspects of ERP are highlighted, along with common obstacles associated with ERP and how to overcome them. The participant then chooses an ERP exercise at the bottom of the exposure hierarchy. *Homework: Start ERP and report to the psychologist after two days*.
Modules 6 - 11 *ERP exercises*	Each module focuses on certain ERP exercises with examples from each treatment character. The text for each module is short (1-2 pages), as the focus is reporting and planning the weekly exposures. *Homework: Conduct daily ERP and report to the psychologist at least once per week*.
Modules 12 - 13 *ERP exercises. Establishing valued directions for further improvements*	The modules focus on daily ERP with further exercises added that are adopted from acceptance and commitment therapy. These include establishing valued based goals and how they are applied in daily exposure tasks. *Homework: Continue ERP. Establish valued based goals and applying them in daily exposure exercises.*
Modules 14 - 15 *Establishing relapse prevention plan*	The treatment is summarized, and the participant learns the distinction between relapse and setback and further treatment strategies. The participant establishes a relapse prevention program based on his/her valued based goals. *Homework: Continue ERP. Sum the treatment and establish a relapse prevention plan.*

The manual was partly tailored to common OCD subtypes (washing, checking, symmetry, and forbidden thoughts). All participants read the same texts relating to general psychoeducation and rationale for the treatment, but specific examples of obsessions and compulsions were given according to the participants' OCD subtype. The participants had to submit homework exercises after each module. One psychologist (EA) treated all participants and provided feedback and support on homework assignments within 36 hours during weekdays. The participants were given consecutive access to the next module after completing quizzes and registrations in the worksheets. The treatment lasted for 15 weeks and included e-mail contact only with the therapist.

### Statistical analysis

SPSS version 19.0 (SPSS inc., Chicago) was used for the statistical analyses. Continuous variables were analyzed by within-group t-tests with Bonferroni corrected alpha levels, ordinal variables were analyzed by Wilcoxon's signed rank test, and nominal variables were analyzed with McNemar's test of change. Cohen's *d *was used to calculate within-group effect sizes using the formula (M_pre _- M_post_)/SD_pooled_. We used the Jacobson & Truax criteria [57] to determine clinical significant improvement using reliable change index with a cutoff of 2 standard deviations below pre-treatment value. We also used the 30% reduction criterion on the Y-BOCS which is a less conservative estimate of clinical improvement; the 30%-criterion has been used in previous studies and has the highest positive efficiency score (91%) of agreeing with CGI change [58]. The sample size was considered satisfactory as power calculations indicated an 87% chance of detecting a significant pre-post difference, given an effect size of 0.6 with an alpha-level of .05.

## Results

### Treatment adherence

The therapist spent a mean time of 92 minutes (SD = 43) per patient during the 15-week treatment (range: 25-203 minutes, median: 80 minutes). The average number of completed modules was 9.74 (SD = 3.93). Some participants had daily contact with the psychologist to report progress with the ERP but a majority of the participants preferred weekly contact. Three participants did not begin the ERP exercises and were therefore considered as dropouts. The reasons for not beginning ERP reported by these participants at post-treatment assessment were lack of time and difficulties getting started with the active treatment. None of the participants reported any changes in medication or parallel psychological treatments at post-treatment assessment.

### Treatment effectiveness

#### Primary outcome

The results are presented in Table 3. Significant reductions in OCD symptoms were found on the primary outcome (Y-BOCS clinician version) (*t *_22 _= 7.88, *p *< .001) with a large effect size of 1.56.

**Table 3:** Means, SDs, Effect Sizes, and p-values of Outcome Measures

Outcome measure		M	SD	Effect size	(CI, 95%)	*p*-value
**Y-BOCS (clin.)**	Pre	20.00	(5.20)			
	
**(n = 23)**	Post	10.00	(7.40)	1.56*	(0.88 - 2.19)	*p *< .001

**Y-BOCS (self-r)**	Pre	20.14	(5.79)			
	
**(n = 22)**	Post	11.50	(7.44)	1.30*	(0.64 - 1.92)	*p *< .001

**OCI-R**	Pre	21.60	(10.08)			
	
**(n = 22)**	Post	9.00	(7.84)	1.39*	(0.72 - 2.02)	*p *< .001

**MADRS-S**	Pre	10.32	(5.61)			
	
**(n = 22)**	Post	4.50	(5.40)	1.06*	(0.41 - 1.67)	*p *< .001

**PSWQ**	Pre	54.41	(12.46)			
	
**(n = 22)**	Post	50.04	(9.79)	0.39	(-0.21 - 0.97)	*p *= .124

**GAF**	Pre	57.87	(7.01)			
	
**(n = 22)**	Post	66.87	(11.83)	0.93*	(0.30 - 1.52)	*p *< .001

**QOLI**	Pre	2.35	(1.42)			
	
**(n = 22)**	Post	2.42	(1.43)	0.05	(-0.54 - 0.63)	*p *= .656

**EQ-5D**	Pre	0.87	(0.19)			
	
**(n = 22)**	Post	0.91	(0.17)	0.24	(-0.35 - 0.83)	*p *= .082

* Significant using Bonferroni corrected alpha values.

Y-BOCS = Yale-Brown Obsessive Compulsive Scales

OCI-R = Obsessive Compulsive Inventory - Revised

MADRS-S = Montgomery Åsberg Depression Rating Scale - Self Report

PSWQ = Penn State Worry Questionnaire

GAF = Global Assessment of Functioning

QOLI = Quality of Life Inventory

EQ-5D = Euroqol

#### Secondary outcomes

**Figure 2:**
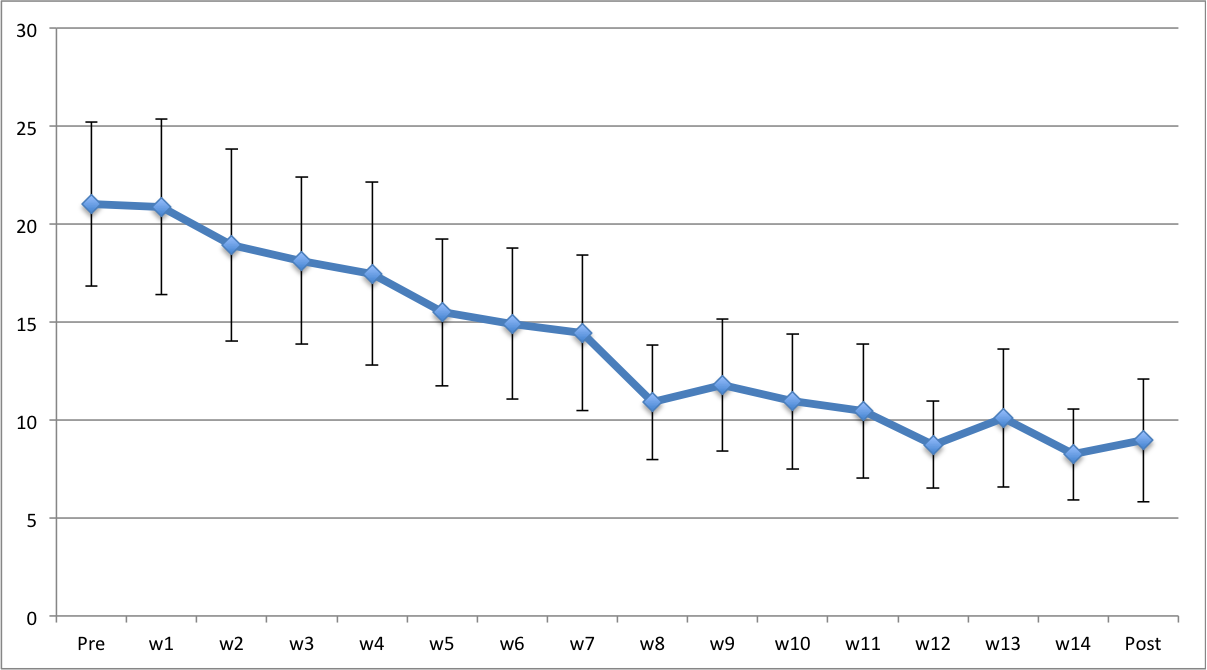
**Means and 95% confidence intervals of weekly OCD symptoms using OCI-R**.

The results are presented in Table 3 and Figure 2. There were significant improvements on the Y-BOCS (self-rating version) (*t *_21 _= 5.93, *p *< .001) and the OCI-R (*t *_21 _= 6.57, *p *< .001) with large effect sizes ranging from 1.30 to 1.39. In addition, there were improvements on GAF (*t *_22 _= 4.40, *p *< .001) and MADRS-S (*t *_21 _= 5.87, *p *< .001) with large effect sizes ranging from 0.93 to 1.06. Results also revealed a non-significant trend (uncorrected) on the EQ-5D (*t *_21 _= 1.83, *p *= .082) with a small effect size of 0.24. The results of the QOLI and PSWQ were non-significant.

#### Clinical significance and CGI

**Figure 3:**
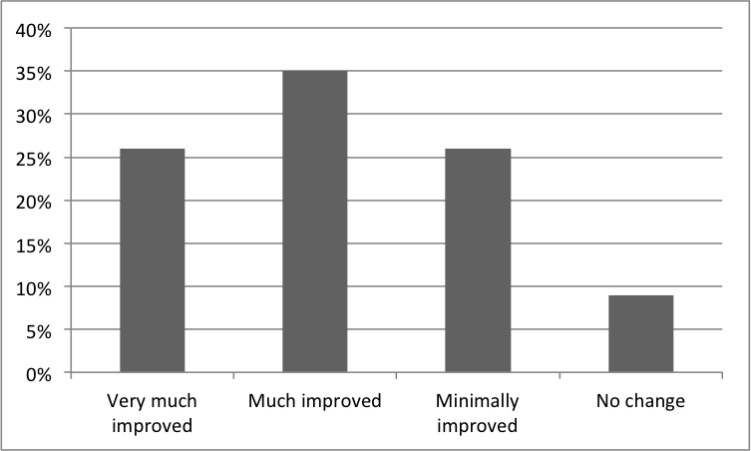
**CGI improvements**.

The number of clinically significant improvements using Jacobson and Truax criteria [57] was 14 of 23 (61%) (*p *< .001). The corresponding figure using the ≥ 30% reduction criterion was 18 of 23 (78%) (*p *< .001) and 10 of the 23 (43%) participants no longer fulfilled the diagnostic criteria of OCD immediately after treatment (*p *< .001). There was a reduction of CGI-severity scores (*Z *= 3.49, *p *< .001); the CGI-improvement scores are presented in Figure 3.

## Discussion

The aim of this open pilot study was to evaluate the effects and feasibility of ICBT for OCD. The results were in line with the previous findings on ICBT with therapist support for anxiety disorders with large within-group effect sizes on the primary outcome measures [59]. The within-group effect sizes for improvements in OCD symptoms were large ranging from 1.30 to 1.56. There were also large effect sizes in general functioning and depression and a small trend towards increased quality of life with EQ-5D. The number of participants meeting diagnostic criteria for OCD after treatment decreased and 61% of the participants reached the criteria for clinically significant improvement. Using the less conservative 30% reduction criterion, the response rate increased to 78%.

The effect sizes in this trial were in the same range as the effects of traditional face-to-face CBT for OCD [5], even though this trial used much less therapist time (92 minutes over the 15-week treatment) than traditional face-to-face treatments, and had higher in-group effect sizes than most trials with strictly computer-based treatments [15,17-20]. One possible explanation for the large effects in this trial was the high accessibility and possibility to have an intensive contact with the therapist during treatment. Intensive contact and increased access to the therapist have been investigated in face-to-face CBT with effect sizes comparable to other trials of CBT for OCD [60]. Furthermore, therapist input has been found to be an important factor in ICBT [21]. The therapist in this trial was very active and had sometimes daily communication with the participants. As a result of this very active therapist input, some participants worked intensively with the treatment and achieved therapeutic benefits (i.e. successful ERP) within a couple of weeks. In addition, the screening and post-treatment assessment interviews were conducted face-to-face by a psychiatrist and this could also have affected the treatment adherence and outcome.

If controlled trials confirm that ICBT with therapist support is indeed effective for OCD, one possible venue for future applications could be to combine face-to-face CBT with Internet support. This would combine two important elements in therapy, the possibility to be flexible using a face-to-face therapist and also opportunity increase and reinforce ERP frequency between the sessions. Other possible applications would be to add weekly videoconference sessions as an adjunct to ICBT. This would in turn expand the treatment arsenal and further increase treatment accessibility for patients suffering from OCD.

This study has several limitations. First, only one psychologist (EA) treated all patients and the limited therapist time achieved in this study is perhaps not fully generalizable to other therapists. However, the time to treat the patients was about 9-10 minutes per module which is in line with previous research studies of ICBT with therapist support [61]. Second, the OCD symptom ratings were lower in this sample than in other trials where Y-BOCS baseline mean ratings range from 24 to 29 [62-64]. One possible consequence is that the effects in this trial are lower because we had less room for Y-BOCS change. Another possible consequence is that this population was easier to treat because they had lower symptom burden, Thus, the lower baseline means could have affected the results in both ways. Third, we excluded patients with extreme OCD (Y-BOCS > 31). As this also affects the generalization of the results, further trials including more severely impaired OCD patients are needed to investigate the effects of ICBT for OCD. Fourth, a majority (57%) of the participants was self-referred and it is possible that we had a very motivated patient population, affecting the generalization of the results. However, Mataix-Cols et al. [65] found that GP-referred patients have best treatment outcome compared to self-referred or mental health professional referred patients. Fifth, a majority of the participants had university education ≥ 3 years, which also could affect the generalization of the results. One interesting future research topic would therefore be to investigate treatment efficacy in ICBT for OCD in relation to both referral type and educational level. Sixth, this study did not include any long-term follow-up data. Our recommendation for future studies is therefore to include follow-up periods and further investigate long-term efficacy of ICBT. Seventh, one of the outcome measures (OCI-R) used in this study has not been validated for online use. Thus, the administration format could have affected the results. Finally, as this was a pilot study, there was no randomization to a control condition, which rendered it difficult to claim any improvements were caused by the treatment alone. Furthermore, the assessors were not blinded to time point. However, OCD is regarded as a stable and often chronic disease [1] and, considering the large effect sizes on both the clinician and self-administered instruments, it is unlikely the overall treatment results could be due spontaneous remission.

## Conclusions

Despite several limitations, the results suggest that ICBT with therapist support has the potential to reduce OCD symptoms, depressive symptoms and general functioning. Controlled trials are needed to further validate ICBT for OCD.

## Competing interests

The authors declare that they have no competing interests.

## Authors' contributions

EA was mainly responsible in designing the study, treating all patients, writing the treatment manual, analyzing data and drafted the manuscript. BL participated in writing the treatment manual and in the drafting of this manuscript. EH participated in the conception of the study and writing the manual. He also contributed to the drafting of the manuscript. BP participated in the conception of the study, writing the manual and in revising this manuscript. VK participated in the study conception, its design and management, analysis of data, interpretation and revising this manuscript. NL participated in the study conception, its design and management, analysis of data, interpretation and revising this manuscript. GA participated in the study conception, its design and management, analysis of data, interpretation and revising this manuscript. CR participated in the conception of the study and its design and in the supervising of this manuscript. All authors read and approved the final manuscript.

## Pre-publication history

The pre-publication history for this paper can be accessed here:

http://www.biomedcentral.com/1471-244X/11/125/prepub
